# Soluble PTX3 of Human Umbilical Cord Blood-Derived Mesenchymal Stem Cells Attenuates Hyperoxic Lung Injury by Activating Macrophage Polarization in Neonatal Rat Model

**DOI:** 10.1155/2020/1802976

**Published:** 2020-01-23

**Authors:** Miyeon Kim, Ji Hye Kwon, Yun Kyung Bae, Gee-Hye Kim, Soyoun Um, Jueun Ha, Soo Jin Choi, Wonil Oh, Hye Jin Jin

**Affiliations:** Biomedical Research Institute, MEDIPOST Co., Ltd., Seongnam 13494, Republic of Korea

## Abstract

Therapeutic treatment of various inflammation-related diseases using mesenchymal stem cells (MSCs) has increased in recent years because of the paracrine action of these cells but shows several limitations. First, MSC-based therapies exhibit varying efficacies; thus, biomarkers should be determined to identify who may benefit from these candidate therapeutic agents. Second, the mechanism underlying the therapeutic effects is poorly understood. To evaluate the effects of human umbilical cord blood-derived MSCs (UCB-MSCs) on macrophages, the macrophage cell line NR8383 stimulated with lipopolysaccharide (LPS) was cocultured by UCB-MSCs. We found that UCB-MSCs mediated changes in macrophage polarization towards M2 from M1 macrophages. To identify the paracrine action underlying the anti-inflammation effect of UCB-MSCs, the secretion of UCB-MSCs exposed to LPS-stimulated NR8383 cells was tested using a biotin label-based 507 antibody array. Among the secreted proteins, we selected pentraxin-related protein PTX3/tumor necrosis factor-inducible gene 14 protein (PTX3) to investigate its association with UCB-MSCs in macrophage polarization. We found that human PTX3 was secreted from UCB-MSCs under inflammation condition and reinforced the M2 macrophage marker via the Dectin-1 receptor by activating MSK1/2 phosphorylation signaling in NR8383 cells. Accordingly, knockdown of PTX3 in UCB-MSCs significantly attenuated their therapeutic effects in a neonatal hyperoxic lung injury resulting in reduced survival, lung alveolarization, M2 marker expression, Dectin-1 levels, anti-inflammatory cytokines, and improved M1 marker expression and inflammatory cytokines compared to control MSC-injected rats. UCB-MSCs show therapeutic potential by controlling macrophage polarization. Interestingly, higher PTX3 levels in UCB-MSCs induced greater improvement in the therapeutic effects than lower PTX3 levels. Collectively, PTX3 is a potential marker with critical paracrine effects for predicting the therapeutic potential of MSC therapy in inflammatory diseases; quality control assessments using PTX3 may be useful for improving the therapeutic effects of UCB-MSCs.

## 1. Introduction

Mesenchymal stem cells (MSCs) exhibit the capacity for continuous self-renewal and for differentiation into specific cells, as well as the ability to regenerate damaged tissues and regulate various immune cell functions [1–4]. Studies have actively focused on the use of MSCs for treating various diseases for the functional recovery of damaged organs or tissues [5–7].

However, some studies have reported lower than anticipated therapeutic effects of MSCs upon injection into damaged tissues. For instance, in a clinical study of intravenous injection of BM-MSCs, only limited clinical effects were observed in patients with severe inflammatory Crohn's disease [8]. Additionally, subcutaneous injection of MSCs in a skin defect model did not lead to wound healing effects [9]. These findings may be attributed to the heterogeneity of MSCs. (1) As they are viable cells, the efficacy of MSCs is predicted to vary according to the situation unlike conventional drugs which display consistent efficacy. (2) Donor variation can lead to variable effects. Thus, the clinical application of MSCs remains limited. Such issues may be resolved by determining criteria for selecting highly efficient stem cells.

Recent studies reported that during inflammation regulation in various diseases, MSCs can induce macrophage polarization, through which therapeutic effects were observed such as suppressed inflammation and enhanced anti-inflammation [10–12]. M1 macrophages are classically activated macrophages; an external stimulus leads to inflammatory actions through the secretion of interleukin- (IL-) 1*α*, IL-6, IL-8, and tumor necrosis factor-*α*. In contrast, M2 macrophages are alternatively activated and cause anti-inflammatory actions by activating IL-4, IL-10, IL-13, and arginase 1 (Arg1). As representative markers, CD11b, CD40, and CD80 are used to identify M1 macrophages, while CD163 and CD206 are used for M2 macrophages [13–15]. Such macrophage polarization recruits key transcription regulatory factors, and modulatory interactions among signal transducer and activator of transcription, interferon regulatory factors, nuclear factor- (NF-) *κ*B, activator protein 1, peroxisome proliferator-activated receptor-*γ*, cAMP response-binding element [13, 16, 17], and others regulate macrophage polarization in various inflammatory diseases [18].

The secretome has been shown to release various therapeutic proteins in response to the microenvironment when MSCs are exposed to an area containing a lesion. Thus, various proteins secreted by MSCs function in recovery of the lesion area, which is referred to as the paracrine effect [19, 20]. Secreted therapeutic proteins are known to act as angiogenic factors, growth and trophic factors, chemokines, and anti-inflammatory cytokines and participate in immune regulation [21]. In particular, according to a previous study, MSCs exposed to an inflammatory condition secrete tumor necrosis factor-inducible gene 6 (TSG6) or prostaglandin E2 (PGE2), which are known to be involved in macrophage polarization [22–24]. However, to explain the therapeutic effects of MSCs based solely on these two proteins, the current understanding of the conversion to anti-inflammatory conditions is insufficient and studies of the signaling mechanism should be needed. It is necessary to identify additional proteins involved in macrophage polarization induced by MSCs and determine the signaling mechanism that enhances anti-inflammation.

In this study, to select highly efficient stem cells effective for treating inflammatory conditions, proteins secreted from MSCs were subjected to mass identification, and PTX3 (PTX3/TSG14, pentraxin-related protein PTX3/tumor necrosis factor-inducible gene 14 protein) was verified as a potential marker. The signaling mechanism by which macrophage polarization is induced was also elucidated based on Dectin-1 receptor-mediated mitogen- and stress-activated protein kinase-1/2 (MSK1/2) phosphorylation of macrophages. Furthermore, the therapeutic effect and mechanism involving PTX3 were tested in an animal model of bronchopulmonary dysplasia, a representative inflammatory disease, and evidence for determining the minimum standards for PTX3 was acquired. The anti-inflammatory therapeutic mechanism of MSCs in inflammatory diseases was established, and we found that the selection criteria for MSCs predicted to exhibit outstanding efficacy may enhance the therapeutic effects of these cells.

## 2. Methods

### 2.1. UCB-MSC Preparation

This study was approved by the Institutional Review Board of MEDIPOST Co., Ltd. (MP-2015-6-4). Umbilical cord blood (UCB) was collected from the umbilical veins after neonatal delivery with informed maternal consent. Harvested UCB was processed within 24 h of collection. UCB-MSCs, isolated and separated from mononuclear cells with Ficoll-Paque™ PLUS (GE Healthcare, Uppsala, Sweden), were washed or suspended in minimum essential medium-alpha (Gibco, Grand Island, NY, USA) supplemented with 10% fetal bovine serum (Gibco). The cultures were maintained at 37°C in a humidified atmosphere containing 5% CO_2_, and the culture medium was changed twice per week [25]. For each passage, MSCs were cultured for 5 days, harvested with trypsin-ethylenediaminetetraacetic acid (Gibco), counted, and then reseeded at a cell density of 2,000 cells/cm^2^. We followed the methods of Jin et al. [26]. In this study, we tested seven UCB-MSC lots that were separated from the UCB samples obtained from different donors; the basic information of these cells is summarized in Supplementary Table 1. In all experiments, UCB-MSCs were used at passage 6. Recombinant PTX3 was obtained from R&D Systems (Minneapolis, MN, USA).

### 2.2. In Vitro Inflammation Condition

The rat alveolar macrophage cell line NR8383 (ATCC, Manassas, VA, USA) was cultured in F-12K medium supplemented with 15% fetal bovine serum. Lipopolysaccharide (LPS, from *Escherichia coli* O55:B5, 1 *μ*g/mL, Sigma-Aldrich, St. Louis, MO, USA) was used to activate the NR8383 cells (1 × 10^5^), which served as a positive control for inflammation conditions. Classically activated NR8383 cells were cocultured with UCB-MSCs (1.9 × 10^4^) for 3 days in a 24-well plate. For paracrine action, cell to cell contact (direct) used similar to clinical application condition. Coculture supernatants were collected, and rat IL-1*α*, rat IL-6, rat IL-8, rat IL-10, and human PTX3 were measured by enzyme-linked immunosorbent assay (ELISA) (all from R&D Systems). For immunofluorescent staining, NR8383 cells were counterstained with Hoechst 33342 (Invitrogen) before coculture and then incubated overnight at 4°C with antibodies against rat CD11b (Abcam, Cambridge, UK), rat CD163 (Santa Cruz Biotechnology, Inc., Santa Cruz, CA, USA), or rat Dectin-1 (Abcam). After three washes, the cells were incubated with Alexa Fluor® 488 or Cy3-conjugated secondary antibody (Jackson ImmunoResearch Europe Ltd., Newmarket, United Kingdom) for 1 h at room temperature in the dark. Fluorescent images were acquired by LSM 800 confocal microscopy (Zeiss, Oberkochen, Germany).

### 2.3. Flow Cytometry

For cytometric analysis of the cultured cell phenotypes, the cells were stained for 15 min at room temperature with fluorescein isothiocyanate-conjugated antibodies against human CD14 and CD45 and human leukocyte antigen-DR isotype (BD Biosciences, Franklin Lakes, NJ, USA); phycoerythrin-conjugated antibodies against human CD73 and CD166 (BD Biosciences) and CD90 and CD105 (Invitrogen). Corresponding isotype-matched mouse antibodies were used as controls. The cells were washed with phosphate-buffered saline (PBS, Corning, Manassas, VA, USA) and fixed with 1% (*v*/*v*) paraformaldehyde (Sigma-Aldrich). The immunotype of the MSCs was determined by flow cytometry on a FACSCalibur instrument (BD Biosciences), and the percentage of expressed cell surface antigens was calculated for 10,000 gated-cell events [26].

### 2.4. Cell Differentiation

The cells were incubated under specific conditions to induce differentiation into osteocytes, chondrocytes, and adipocytes, and their multilineage potential was evaluated as previously described [27]. Briefly, osteoblast or osteocyte formation was assessed by measuring the activity of alkaline phosphatase (Sigma-Aldrich). To confirm chondrogenic differentiation, cryosections were analyzed by Safranin O staining (Sigma-Aldrich). Adipocyte formation was assessed based on the staining of accumulated lipid vacuoles with Oil red O (Sigma-Aldrich) [26].

### 2.5. Secrete Array

We prepared conditioned media from each group (NR8383 cells alone or LPS-treated NR8383 cells; UCB-MSCs alone or UCB-MSCs with LPS-treated NR8383 cells). Protein expression screening was conducted using the Label-Based Human Antibody Array, a differential screening antibody microarray (RayBiotech, Peachtree Corners, GA, USA) which contains 507 antibodies (Supplementary Table 2). The antibody array experiment was performed by E-biogen (Seoul, Korea), according to an established protocol. Fluorescence detection were conducted using a GenePix 4000B (Axon Instruments, Union City, CA, USA) until the glass chips were completely dry, and the chips were scanned on a GenePix ×4000 scanner (GenePix 4000B, Axon Instruments) and the images were analyzed with GenePix Pro 6.0 software (Axon Instruments). After subtracting the background signals and normalizing the values to the positive controls, signal intensities between and among array images were compared to determine the relative differences in expression levels of each protein between groups (UCB-MSCs alone vs. UCB-MSCs with LPS-treated NR8383 cells).

### 2.6. Quantitative Real-Time Polymerase Chain Reaction (qPCR) and Small Interfering RNA (siRNA)

qPCR was performed using a LightCycler™ 480 System (Roche, Basel, Switzerland). TaqMan probes were designed with the Universal Probe Library Assay Design Center (Roche; see Supplementary Table 3) and used to quantitatively detect the mRNA levels of the PTX3 and Dectin-1 genes. The relative expression levels of these mRNAs were calculated using the comparative threshold cycle method (2^−*ΔΔ*Ct^) with normalization to the *β*-actin mRNA expression level [26]. Dharmacon (Lafayette, CO, USA) designed the PTX3 siRNA and scrambled siRNA for use in the siRNA experiments. siRNAs were transfected using DharmaFECT 1 Transfection Reagent (Dharmacon) according to the manufacturer's instructions. The siRNA pools consisted of four different siRNA duplexes (see Supplementary Table 3).

### 2.7. Animal Model

All animal experiments were approved by the Institutional Animal Care and Use Committee of MEDIPOST Co., Ltd. (MP-LAR-2015-12-1). To prepare a bronchopulmonary dysplasia (BPD) *in vivo* model, timed pregnant Sprague-Dawley rats (Samtako Bio Korea Co. Ltd., Osan, Korea) spontaneously delivered newborn rat pups as previously reported [28]. Two experimental designs were used (Supplementary Table 4). Normoxic rats were maintained in normal room air, whereas hyperoxic rats were raised in hyperoxic chambers (90% oxygen) from birth until postnatal day (P) 14. Nursing mother rats were rotated daily between the hyperoxia and room air litters to prevent oxygen toxicity. On postnatal day (P) 5, UCB-MSCs were transplanted intratracheally [29]. An equal volume of PBS was injected intrathecally as a control. The survival rate was daily recorded until the 14th day after birth. On P14, the rat pups were sacrificed under deep pentobarbital anesthesia (60 mg/kg, intraperitoneal), and lung tissue was harvested for morphometric and biochemical analyses. Fixed lung tissues were embedded in paraffin and sectioned to 4 *μ*m thickness followed by staining with hematoxylin and eosin (H&E). The degree of alveolarization was measured using the mean linear index (MLI) as previously introduced [29]. A minimum of three sections per rat and 100 fields per section were randomly assessed in a blinded manner. Engraftment of the infused MSCs was measured by immunofluorescence analysis of human *β*2 microglobulin (*β*2MG, Santa Cruz) visualized using an Alexa Fluor® 488-labeled secondary antibody (Jackson ImmunoResearch Europe Ltd.). To detect alveolar macrophages, primary antibodies were used to detect CD11b, CD163, and Dectin-1 in the lung sections. The slides were incubated with primary antibody overnight at 4°C. Secondary antibodies were stained with Alexa Fluor® 488 or Cy3 (Jackson ImmunoResearch Europe Ltd.) for 1 h at room temperature. Nuclei were counterstained with Hoechst 33342. Section images were acquired by confocal microscopy. The concentrations of rat IL-6, rat IL-8, and rat IL-10 in the bronchoalveolar lavage fluid (BALF) were tested by ELISA as previously described [28, 29]. The basic scheme of the rat model is summarized in Supplementary Figure 1.

### 2.8. Statistical Analyses

All data are reported as the mean ± standard deviation and were analyzed with SPSS software (version 18; SPSS, Inc., Chicago, IL, USA). Significant differences were verified by one-way analysis of variance followed by the least-significant difference post hoc test. Student's *t*-test was used to compare data between two groups. *P* values less than 0.05 were considered statistically significant.

## 3. Results

### 3.1. UCB-MSCs Regulate Macrophage Polarization in In Vitro Inflammation Model

**Figure 1:**
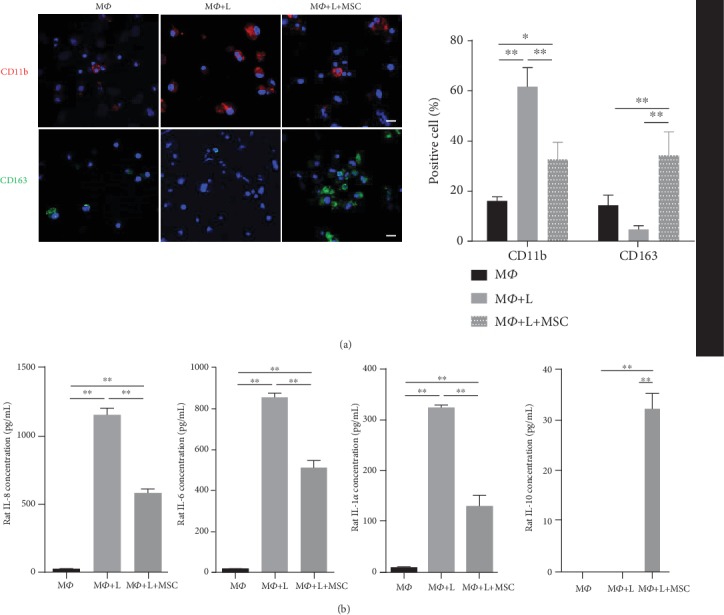
Macrophage polarization effect of UCB-MSCs under LPS-induced inflammation conditions. Rat alveolar macrophages (NR8383) were stimulated with LPS and cocultured with UCB-MSCs for 3 days. (a) The macrophage markers M1 and M2 were examined by staining with CD11b or CD163, respectively. Expression levels of CD11b (red) and CD163 (green) were analyzed by confocal microscopy. Nuclei were stained with Hoechst 33342 (blue). Quantitative results are displayed as the percentage of CD11b- or CD163-positive cells. Scale bar = 100 *μ*m. (b) Cell supernatants were analyzed for inflammatory cytokines (rat IL-1*α*, rat IL-6, and rat IL-8) or anti-inflammatory cytokine (rat IL-10) by ELISA. (a, b) Error bars represent the means ± SD, *n* = 5 per group; ^∗∗^*P* < 0.01, ^∗^*P* < 0.05. M*Ф*: macrophage; L: LPS.

MSCs are widely known for their anti-inflammation actions, which occur through cell-to-cell contact, the activities of soluble factors including growth factors, cytokines, matrix inhibitors, and extracellular vesicles, or a combination of these mechanisms [30]. Previous studies showed that MSCs mediated changes in macrophage polarization to alternatively activate M2 macrophages from M1 macrophages, likely contributing to inflammation reduction [13–15]. Macrophages are polarized towards proinflammatory (M1) or anti-inflammatory (M2) macrophages. M1 macrophages induce local inflammation by releasing proinflammatory cytokines (IL-1, IL-6, IL-8, TNF-*α*, or IFN-*γ*), while M2 macrophages secrete anti-inflammatory cytokines (IL-10 and TGF-*β*1) which have anti-inflammatory effects and enable tissue regeneration following inflammation conditions [16]. M1 and M2 polarization resulted in distinct surface marker profiles with high expression of CD86 and high levels of CD11b or CD163 and the mannose-binding lectin receptors CD206 and CD209 on M2 macrophages [17, 18]. Here, to analyze the macrophage polarization effect of UCB-MSCs, rat alveolar macrophages (NR8383 cells) stimulated by LPS were cocultured with UCB-MSCs. After 72 h of culture, NR8383 was stained with M1 proinflammatory marker (CD11b) or M2 anti-inflammatory marker (CD163), and then, the number of positive cells was counted. CD11b was activated in LPS-stimulated NR8383 cells; however, this was significantly blocked by coculture with UCB-MSCs. CD163 expression of NR8383 cells was significantly increased during coculture (Figure 1(a)). Next, we analyzed the levels of proinflammatory cytokines (rat IL-1*α*, rat IL-6, and rat IL-8) and an anti-inflammatory cytokine (rat IL-10) in NR8383 cells. Secretion of proinflammatory cytokines was elevated following LPS induction, while these increases were significantly inhibited by coculture with UCB-MSCs. The anti-inflammatory cytokine secretion from NR8383 cells cultured with UCB-MSCs was significantly higher than that from NR8383 cells alone (Figure 1(b)). These results demonstrate that UCB-MSCs promote macrophage polarization *in vitro* under inflammation conditions.

### 3.2. Identification of Secreted Proteins from UCB-MSCs Stimulated under Inflammation Conditions

**Figure 2:**
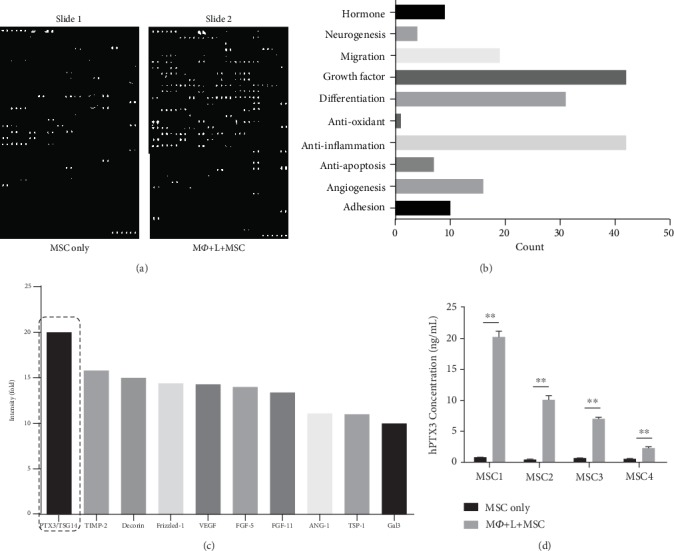
Secretion protein array of UCB-MSCs with altered expression under inflammation conditions. (a) Biotin label-based antibody array analysis using conditioned medium collected from UCB-MSCs alone versus UCB-MSCs cocultured with LPS-treated NR8383 cells. Proteins secreted under each condition evaluated with a proteome profiler. (b) For the 192 proteins upregulated in UCB-MSCs treated with LPS-induced NR8383 cells, functional categories were classified by biological process annotation. (c) Quantification of the optical intensity of anti-inflammation factor. Intensity analysis showed upregulated protein secretion in the UCB-MSC coculture system compared to the UCB-MSCs cultured alone. Protein levels were evaluated as the fold increase, with data normalized to intensity of UCB-MSCs alone, which was defined as 1. PTX3 showed the most significant increase in the UCB-MSC coculture system (black box). (d) To confirm the upregulation of PTX3, PTX3 secretion level was measured in 4 different samples by ELISA for UCB-MSCs alone and for cocultured UCB-MSCs. Error bars represent the means ± SD, *n* = 3 per group; ^∗∗^*P* < 0.01. M*Ф*: macrophage; L: LPS.

While the concept of macrophage polarization may be explained by MSCs, the biological relevance of these finding remains unclear. Various soluble proteins have been proposed to govern the beneficial effects of MSCs, such as their immune modulation and anti-inflammation effects [31]. Thus, we hypothesized that UCB-MSCs use secreted proteins to actively control macrophage polarization. To test this hypothesis, we analyzed the proteins secreted from UCB-MSCs to identify paracrine factors associated with macrophage polarization effects using the 507 biotin label-based antibody array with conditioned media from each group (NR8383 cells alone or LPS-treated NR8383 cells; UCB-MSCs alone or UCB-MSCs with LPS-treated NR8383 cells). No changes in the expression of secreted proteins was observed in NR8383 cells with and without LPS stimulation (data not shown), which we analyzed compared with UCB-MSCs following in the presence or absence of LPS stimulated NR8383 (Figure 2(a)). Proteins with a ratio of more than 2.0 were considered to show increased section from UCB-MSCs when cocultured with LPS-stimulated NR8383 cells. Upregulated proteins in cocultured UCB-MSCs compared to UCB-MSCs cultured alone were related to 192 functional biological pathways including regulation of hormones, neurogenesis, migration, growth factor, differentiation, anti-oxidant, anti-inflammation, anti-apoptosis, angiogenesis, and adhesion (Figure 2(b)). Among the upregulated proteins in biological processes, we analyzed the increases in proteins involved in anti-inflammation signaling. We selected 10 secreted proteins that were markedly upregulated in UCB-MSCs following coculture, PTX3, TIMP-2, Decorin, Frizzled-1, VEGF, FGF-5, FGF-11, Ang-1, TSP-1, and Gal3, by intensity analysis (Figure 2(c)). Notably, PTX3 showed the greatest upregulation under inflammation-stimulation conditions. To verify these array results, we analyzed the expression levels of PTX3 in UCB-MSCs and cocultured UCB-MSCs from an additional 4 different donors by ELISA. The level of PTX3 was increased under inflammation conditions in all four UCB-MSC lots tested, with increases observed in the secretion of MSC1 (10-fold), MSC2 (9-fold), MSC3 (3.5-fold), and MSC4 (3-fold), as shown in Figure 2(d). Taken together, we selected PTX3 as a potential marker to further investigate its involvement in the inflammation of UCB-MSCs.

### 3.3. PTX3 of UCB-MSCs Increases Anti-Inflammation Effect through Macrophage Polarization

**Figure 3:**
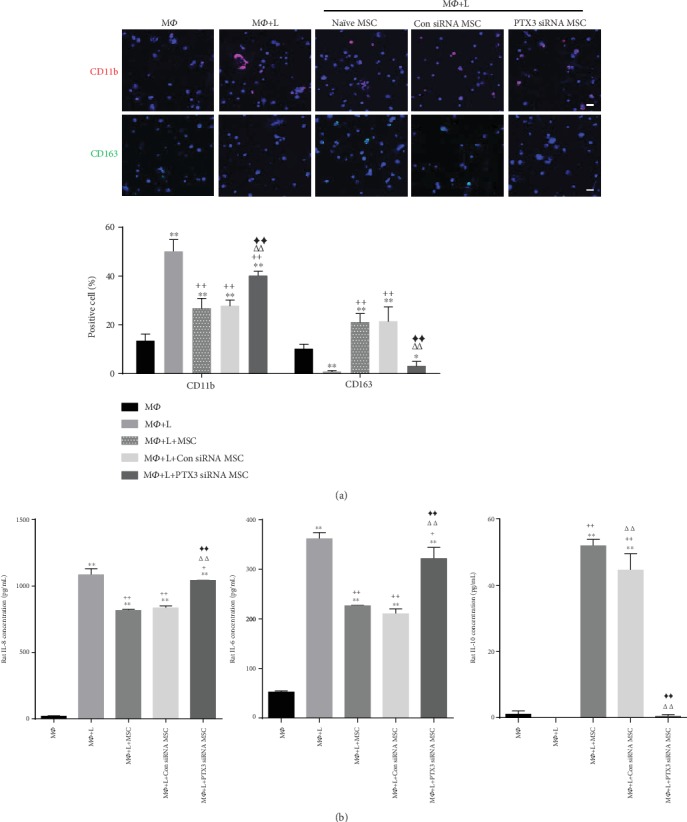
PTX3 knockdown in UCB-MSCs accelerates macrophage polarization. NR8383 cells were stimulated with LPS and cocultured with UCB-MSCs for 3 days. UCB-MSCs were pretreated with control siRNA or PTX3 siRNA before coculture. (a) Expression of CD11b (red) and CD163 (green) was assessed by quantifying the percentage of positively stained cells. Nuclei were stained with Hoechst 33342. Scale bar = 100 *μ*m. (b) Cell supernatants were analyzed for inflammatory cytokines (rat IL-6, rat IL-8) or anti-inflammatory cytokine (rat IL-10) by ELISA. (a, b) Error bars represent the means ± SD, *n* = 5 per group; ^∗^*P* < 0.05, ^∗∗^*P* < 0.01 vs. M*Ф*. ^+^*P* < 0.05, ^++^*P* < 0.01 vs. M*Ф*+L. *^ΔΔ^P* < 0.01 vs. M*Ф*+L+naïve MSC. ^◆◆^*P* < 0.01 vs. M*Ф*+L+Con siRNA MSC. M*Ф*: macrophage; L: LPS.

To determine whether PTX3 secretion from UCB-MSCs functionally contributed to macrophage polarization, we blocked PTX3 expression using a siRNA. Control experiments showed that treatment with the target siRNA effectively reduced PTX3 expression at the secretion level (Supplementary Figure 2). On day 3 after coculture, to evaluate macrophage polarization, NR8383 cells were stained with M1 proinflammatory marker (CD11b) or M2 anti-inflammatory marker (CD163), and the number of positive cells was counted. Treatment with siRNA for PTX3 in UCB-MSCs significantly activated CD11b expression, while CD163 expression was largely reduced compared to naïve cells or those treated with control siRNA (Figure 3(a)). At the cytokine level, PTX3 siRNA cells showed increased secretion of inflammatory cytokines (rat IL-6 or rat IL-8), whereas the level of anti-inflammatory cytokine (rat IL-10) was attenuated compared to that in the control group (naïve or control siRNA) (Figure 3(b)). Moreover, to examine the causative factor of PTX3 action under inflammation conditions, we added 100 ng/mL of recombinant PTX3 protein during LPS stimulation of NR8383 cells for 72 h. As expected, PTX3 treatment decreased CD11b expression and increased CD163 expression. Additionally, proinflammation was significantly decreased in the treated groups, which showed prominent secretion of anti-inflammatory molecules (Supplementary Figure 3). Taken together, these results demonstrate that PTX3 secreted as a part of paracrine action plays an important role in macrophage polarization by UCB-MSCs under inflammation conditions.

### 3.4. PTX3 Augments the Macrophage Polarization Effect by Activating Dectin-1 Downstream Signaling

**Figure 4:**
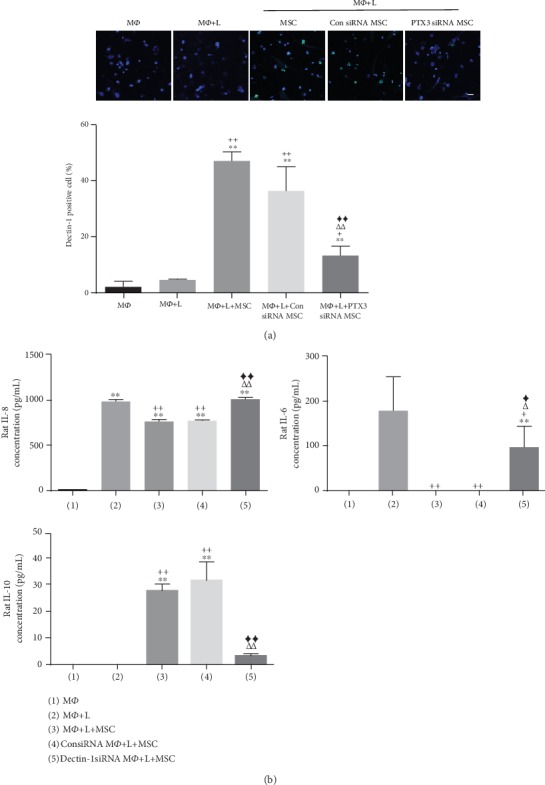
Dectin-1 of macrophages was activated by PTX3 of UCB-MSCs under inflammation condition. (a) NR8383 cells were stimulated with LPS and cocultured with UCB-MSCs for 3 days. UCB-MSCs were pretreated with control siRNA or PTX3 siRNA before coculture. The expression of Dectin-1 (green) in NR8383 cells was assessed by quantifying the percentage of positively stained cells. Nuclei were stained with Hoechst 33342. The merged image is an overlay of the Hoechst 33342 and Dectin-1 images. Scale bar = 100 *μ*m. Error bars represent the means ± SD, *n* = 5 per group; ^∗∗^*P* < 0.01 vs. M*Ф*. ^+^*P* < 0.05, ^++^*P* < 0.01 vs. M*Ф*+L. *^ΔΔ^P* < 0.01 vs. M*Ф*+L+naïve MSC. ^◆◆^*P* < 0.01 vs. M*Ф*+L+Con siRNA MSC. (b) NR8383 cells were stimulated with LPS and cocultured with UCB-MSCs for 3 days. NR8383 cells were pretreated with control siRNA or Dectin-1 siRNA before coculture. Cell supernatants were analyzed for inflammatory cytokines (rat IL-6, rat IL-8) or anti-inflammatory cytokine (rat IL-10) by ELISA. Error bars represent the means ± SD, *n* = 5 per group; ^∗∗^*P* < 0.01 vs. M*Ф*. ^+^*P* < 0.05, ^++^*P* < 0.01 vs. M*Ф*+L. *^Δ^P* < 0.05, *^ΔΔ^P* < 0.01 vs. M*Ф*+L+MSC. ^◆^*P* < 0.05, ^◆◆^*P* < 0.01 vs. Con siRNA M*Ф*+L+MSC. M*Ф*: macrophage; L: LPS.

To investigate the signal pathway of PTX3-induced macrophage polarization, we evaluated whether PTX3 interacts with its known receptor. Although the PTX3-specific cellular receptor is not well-known, recent studies showed that macrophages stimulated with PTX3 expressed higher levels of Dectin-1 [32], which may be an indirect action of PTX3. We evaluated the correlation between PTX3 secretion and Dectin-1 in macrophages by inducing inflammatory conditions. Dectin-1 levels in macrophages were higher in the cocultured UCB-MSCs (naïve or control siRNA) but were significantly downregulated in PTX3 siRNA-treated cells (Figure 4(a)). To examine whether Dectin-1 expression functionally contributed to macrophage polarization, we inhibited Dectin-1 expression in NR8383 cells with siRNA. Control experiments showed that treatment with the target siRNA effectively suppressed Dectin-1 expression at the gene level (Supplementary Figure 4). At day 3 after coculture, to evaluate macrophage polarization, treatment with Dectin-1 siRNA in macrophages enhanced inflammatory cytokine (rat IL-6, rat IL-8) release and reduced anti-inflammatory cytokine (rat IL-10) secretion (Figure 4(b)). Notably, silencing of Dectin-1 affected macrophage polarization. The importance of Dectin-1 was further demonstrated by the result showing that treatment with PTX3 recombinant protein increased Dectin-1 levels (Supplementary Figure 4). These results clearly suggest that Dectin-1 plays a main role in macrophage polarization which triggers the anti-inflammatory effects of PTX3 by UCB-MSCs. Next, we investigated Dectin-1 downstream signaling. The protein kinases MSK1/2 are known to be crucial modulator that limit inflammatory cytokines and increase anti-inflammatory cytokine production by macrophages in response to Dectin-1 [33]. Moreover, it was reported that Dectin-1 induces M2 macrophage polarization by provoking MSK1/2 [33]. To examine whether the PTX3 and Dectin-1 signaling cascade uses a similar mechanism, we examined whether PTX3 or Dectin-1 could mediate each other's expression. We suppressed PTX3 or Dectin-1 by using siRNA in UCB-MSCs and NR8383 cells. On day 3 of coculture, MSK1/2 phosphorylation was increased in NR8383 cells cocultured with control UCB-MSCs (naïve or control siRNA). However, MSK1/2 expression was significantly reduced when the UCB-MSCs were treated with PTX3 siRNA. Additionally, MSK1/2 levels were significantly decreased in NR8383 cells treated with Dectin-1 siRNA (Supplementary Figure 5a and 5b). Importantly, treatment with SA747651A, a chemical inhibitor of MSK1/2, significantly abrogated the decrease in rat IL-8 and increase in rat IL-10 secreted by NR8383 cocultured with UCB-MSCs (Supplementary Figure 5c). These data suggest that PTX3 in UCB-MSCs plays an important role by controlling the Dectin-1 receptor on macrophages, which induces macrophage polarization via MSK1/2 signaling.

### 3.5. PTX3 Improves the Therapeutic Capacity of UCB-MSCs in a Hyperoxic Lung Injury Rat Model

**Figure 5:**
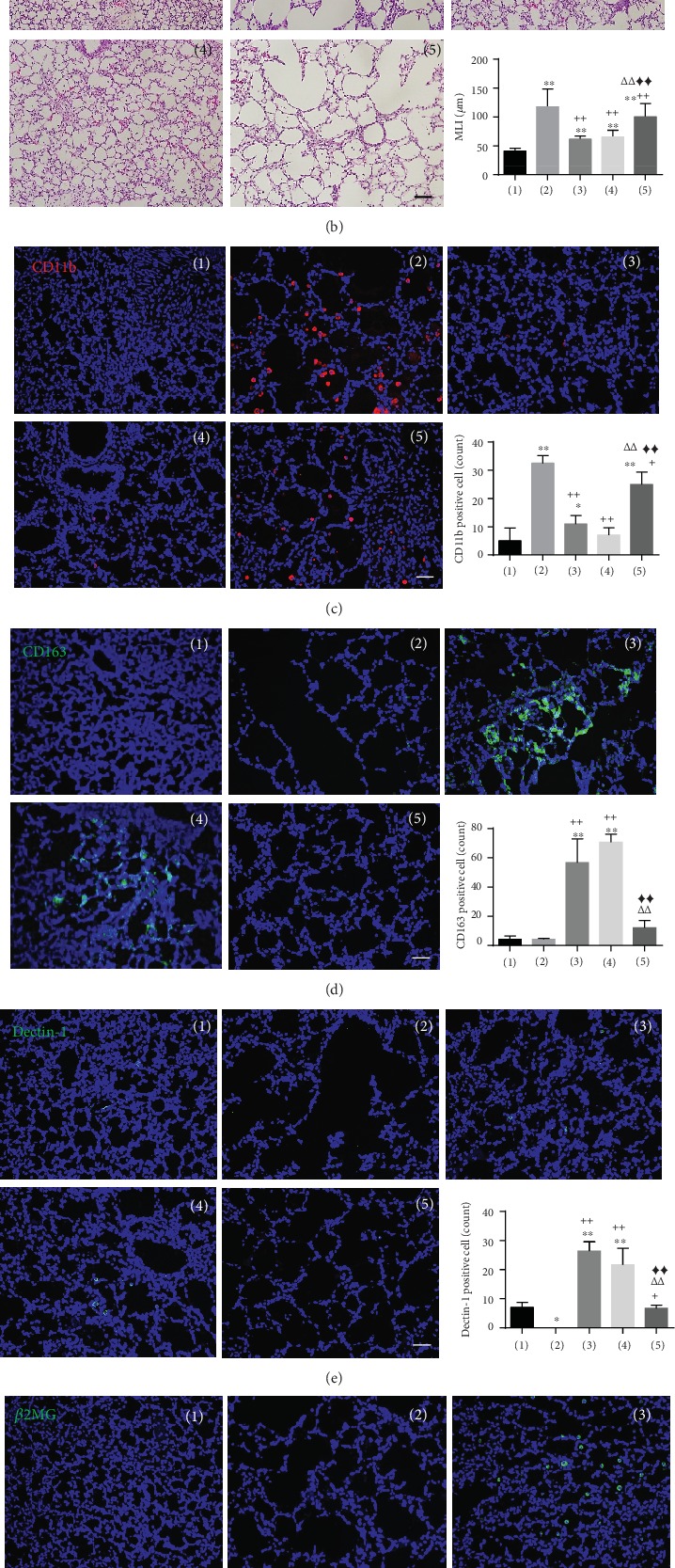
Reduced therapeutic effect of UCB-MSCs by PTX3 knockdown in a rat hyperoxic lung injury model. Normal rats maintained in normoxia room air, whereas hyperoxic rats were raised in hyperoxic chambers (90% oxygen) from birth until P14 and then intratracheally injected with MSCs (naïve, control siRNA MSC, and PTX3 siRNA). Nine days after cell treatment, lung tissue and lung BALF were collected. (a) Survival rate for 14 days after birth. (b) Histologic and morphometric analysis of rat lung tissue. Representative microscopy photomicrographs of lung tissue with hematoxylin and eosin (H&E) staining. Scale bar = 100 *μ*m. Degree of alveolarization shown as the mean linear intercept (MLI, *μ*m). Error bars represent the means ± SD, *n* ≥ 100 per group; ^∗∗^*P* < 0.01 vs. normal. ^++^*P* < 0.01 vs. BPD. *^ΔΔ^P* < 0.01 vs. BPD+naïve MSC. ^◆◆^*P* < 0.01 vs. BPD+Con siRNA MSC. (c, d) Representative immunofluorescence staining using macrophage markers (CD11b, CD163) in rat lung tissue. Expression of CD11b (c, red) and CD163 (d, green) was counted as positively stained cells. (e) Expression of Dectin-1 (green) in lung tissue was counted as positively stained cells. (f) Immunohistochemical detection of human *β*2MG (green) in lung tissue. (c–f) Nuclei stained with Hoechst 33342. Scale bar = 100 *μ*m. (g) Secretion levels of rat IL-6, rat IL-8, and rat IL-10 in the lung BALF. (c–g) Error bars represent the means ± SD, *n* = 5 per group; ^∗^*P* < 0.05, ^∗∗^*P* < 0.01 vs. normal. ^+^*P* < 0.05, ^++^*P* < 0.01 vs. BPD. *^Δ^P* < 0.05, *^ΔΔ^P* < 0.01 vs. BPD+naïve MSC. ^◆^*P* < 0.05, ^◆◆^*P* < 0.01 vs. BPD+Con siRNA MSC. BPD: bronchopulmonary dysplasia; hyperoxic lung injury.

We investigated the significance of the ability of PTX3 to control anti-inflammation effects in an *in vivo* model. We prepared different types of UCB-MSCs including naïve, control siRNA, and PTX3 siRNA and then injected these cells into a severe rat hyperoxic lung injury model (BPD) at P5 and compared the therapeutic outcomes. We observed decreased PTX3 secretion in the PTX3 siRNA group, with suppression maintained for up to 13 days (Supplementary Figure 6a). Nine days after MSC transplantation at P14, the human PTX3 proteins were detected in the control group (naïve or control siRNA) but not in the PTX3 siRNA MSC group (Supplementary Figure 6b). The survival rates were that 47.8% of rats in the BPD group had died, while most rats transplanted with naïve MSCs (77.3%), control siRNA MSCs (82.3%), or PTX3 siRNA MSCs (68.1%) survived (Figure 5(a)). In the rat lung tissue, representative photomicrographs revealed histopathological differences which were confirmed in morphometric analysis by the MLI. Impaired alveolar growth was observed as fewer alveoli or larger alveoli in the BPD group compared to that in the normal group. Hyperoxic lung injury represented by a high MLI was reduced in both the naïve MSC and Con siRNA MSC groups but not in the PTX3 siRNA MSC group (Figure 5(b)). The results of immunofluorescence staining to detect the M1 inflammatory marker (CD11b) or M2 anti-inflammatory marker (CD163) of macrophage polarization were quantified by counting the number of positive cells in the rat lung tissue. CD11b was activated in the BPD group; however, this was significantly blocked by injection of MSCs in the control group (naïve or control siRNA). CD163 expression in the BPD model was significantly enhanced following treatment with the MSC control. As expected, the PTX3 siRNA-treated group showed increased CD11b expression and decreased CD163 expression compared to the MSC control group (Figures 5(c) and 5(d)). Additionally, Dectin-1-positive cells were significantly decreased in the PTX3 siRNA group (Figure 5(e)). When we analyzed the number of engrafted cells by staining of the lung tissue with an antibody specific to human *β*2MG, PTX3 siRNA showed smaller engraftment potency in the lung than in the control group (Figure 5(f)). Next, we measured cytokine expression levels in the lung BALF. The level of inflammatory cytokines including rat IL-6 and rat IL-8 was significantly upregulated in the BPD model compared to normal rats. Hyperoxia stimulated increased inflammatory cytokine release in the BPD rats, which was generally significantly decreased in the control MSC group but not in the PTX3 siRNA group. The anti-inflammatory cytokine level (rat IL-10) in BALF in BPD rats was more effectively reduced by PTX3 siRNA MSC than by treatment with naïve MSCs or control siRNA-treated MSCs (Figure 5(g)). These results demonstrate that PTX3 secretion plays an important role in the macrophage polarization effect in the UCB-MSC *in vivo* disease model. Taken together, these data suggest that inhibiting PTX3 expression accelerated the therapeutic effect of MSCs for treating BPD.

**Figure 6:**
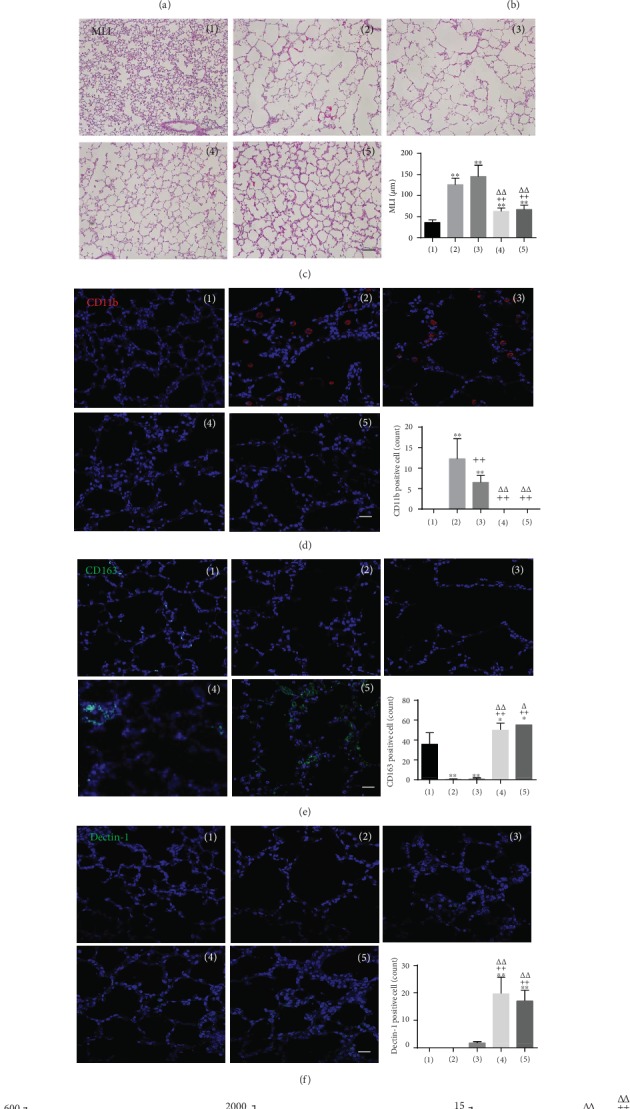
Comparison of therapeutic outcome by PTX3 secretion level using hyperoxic lung injury rat model. (a) NR8383 cells were stimulated with LPS and cocultured with 3 lots of UCB-MSCs for 3 days. PTX3 secretion significantly differed under inflammation conditions following treatments. Error bars represent the means ± SD, *n* = 3 per group; ^∗∗^*P* < 0.01 vs. M*Ф*. ^++^*P* < 0.01 vs. M*Ф*+L. *^ΔΔ^P* < 0.01 vs. M*Ф*+L+MSC5. ^◆◆^*P* < 0.01 vs. M*Ф*+L+MSC6. (b) Daily survival rate during14 days after birth. (c) Histologic and morphometric analysis of rat lung tissue. Representative microscopy photomicrographs of lung tissue with H&E staining. Scale bar = 100 *μ*m. Degree of alveolarization analyzed by MLI. Error bars represent the means ± SD, *n* ≥ 100 per group; ^∗∗^*P* < 0.01 vs. normal. ^++^*P* < 0.01 vs. BPD. *^ΔΔ^P* < 0.01 vs. BPD+MSC5. (d, e) Representative immunofluorescence staining using macrophage markers (CD11b, CD163) in lung tissue. Expression of CD11b (d, red) and CD163 (e, green) was counted as positively stained cells. (f) Expression of Dectin-1 (green) in lung tissue was counted as positively stained cells. (d–f) Nuclei were stained with Hoechst 33342. Scale bar = 100 *μ*m. (g) Secretion levels of rat IL-6, rat IL-8, and rat IL-10 in the lung BALF. (d–g) Error bars represent the means ± SD, *n* = 5 per group; ^∗^*P* < 0.05, ^∗∗^*P* < 0.01 vs. normal, ^++^*P* < 0.01 vs. BPD, *^ΔΔ^P* < 0.01 vs. BPD+MSC5, ^◆^*P* < 0.05, ^◆◆^*P* < 0.01 vs. BPD+MSC6. M*Ф*: macrophage; L: LPS; BPD: bronchopulmonary dysplasia.

In a different set of experiments, we compared the therapeutic effect of PTX3 secretion using a hyperoxic lung injury model in neonatal rat. The three MSC lots were used: MSC5 was defined as the low lot (1.5 ng/mL), MSC6 was defined as the medium lot (6 ng/mL), and MSC7 was used as the high lot (20 ng/mL). To evaluate the human PTX3 secretion level *in vitro* under inflammation conditions, LPS-stimulated NR8383 cells were cocultured with UCB-MSCs (Figure 6(a)). The MSC5, MSC6, and MSC7 lots were prepared and used to treat a severe rat hyperoxic lung injury model at P5 followed by analysis of their therapeutic effects. At day 9 after cell injection, the survival rates were 53.6% in the BPD group, 60% in the MSC5 group, 86.6% in the MSC6 group, and 72.2% in the MSC7 group, as shown in Figure 6(b). The level of alveolarization was analyzed by H&E staining and by measuring the MLI. Hyperoxic lung injury significantly represented by a high MLI was reduced in both MSC6 and control MSC7 but not in the MSC5 group (Figure 6(c)). For macrophage polarization, lung tissue was stained with M1 marker (CD11b) or M2 marker (CD163) and then quantified by counting the number of positive cells. CD11b was activated in the BPD group, which was significantly decreased by treatment with the three lots of MSCs, while injection with MSC6 or MSC7 effectively decreased CD11b levels (Figure 6(d)). CD163 expression was significantly activated following treatment with MSC6 or MSC 7; however, these effects were not observed following injection with MSC5 (Figure 6(e)). Additionally, the Dectin-1 level in cells treated with MSC6 or MSC7 was significantly higher than that in cells treated with MSC5 (Figure 6(f)). Next, we analyzed proinflammatory cytokines (rat IL-6, rat IL-8) and an anti-inflammatory cytokine (rat IL-10) in the rat lung BALF. Secretion of proinflammatory cytokines was elevated in the BPD rats; however, these effects were significantly inhibited by injection with the 3 lots of MSCs. In detail, rat IL-6 showed that MSC6 had significantly higher effect than MSC5 or MSC7. The results of rat IL-8 showed that MSC7 had significantly greater effects than MSC5 and MSC6. The secretion of anti-inflammatory cytokines was significantly increased by both MSC6 and MSC7 but not by MSC5 (Figure 6(g)). These results suggest that the level of PTX3 secretion determines the outcome of treatment of the lung damage model. Taken together, these findings demonstrate that PTX3 protein enhances the therapeutic potency of MSCs for treating BPD.

## 4. Discussion

In this study, UCB-MSCs suppressed inflammation and enhanced anti-inflammation through the actions of PTX3 protein and affected macrophage polarization, in both *in vitro* and *in vivo* models of inflammation.

When UCB-MSCs were cocultured with LPS-stimulated macrophages, a significant decrease in inflammatory cytokines and marked increase in anti-inflammatory cytokines were observed. Similarly, the M1 marker, CD11b, was significantly reduced, and the M2 marker, CD163, was activated. According to a recent report, secretion of protein by MSCs is involved in inducing macrophage polarization, suppressing inflammation, and enhancing anti-inflammation [24]. We analyzed the 507 secretome array using cell culture medium based on the prediction that the proteins secreted by UCB-MSCs in an inflammatory condition induce macrophage polarization. Compared to the medium of UCB-MSCs alone, the levels of 192 proteins were increased by over 2-fold in the medium of UCB-MSCs cocultured with LPS-stimulated macrophages. These proteins were categorized as being involved in bioprocessing via hormones, neurogenesis, migration, growth factors, differentiation, anti-oxidant, anti-inflammation, anti-apoptosis, angiogenesis, and adhesion. Ten proteins involved in anti-inflammation were selected as candidates (PTX3, TIMP-2, Decorin, Frizzled-1, VEGF, FGF5, FGF-11, ANG-1, TSP-1, and Gal3). TSG6, which has been frequently examined in previous studies [22, 23, 34], was excluded because it was increased by less than 2-fold. PTX3 exhibited the largest increase and thus was selected as a potential marker. Analysis of the four lots confirmed a distinct increase in PTX3 levels in all coculture media despite differences in the rate of increase. Other protein candidates require further analysis. VEGF, TSP-1, and Gal3 secreted by UCB-MSCs are known to have therapeutic effects in animal models of various diseases [35–37]. Thus, additional markers may be identified in further studies. Additionally, the paracrine factors secreted by MSC were also regulated by mediators Nuck and Rap1 to control the proinflammatory cytokines [38–40]. The regulation of paracrine effects and secretion factors of MSCs was important to determine the therapeutic efficiency of MSCs.

PTX3 has been reported to induce the polarization of macrophages into anti-inflammatory M2, while stimulating them to secrete immunosuppressive cytokines such as TGF-*β* and IL-10 [41, 42]. For instance, when PTX3-silenced apoptotic macrophages with artificially reduced PTX3 levels were treated with recombinant PTX3, inflammatory cytokines were significantly reduced and anti-inflammatory cytokines were activated [43]. These reports suggest that PTX3 is a key factor in suppressing inflammation and enhancing anti-inflammation. Based on this, PTX3 knockdown UCB-MSCs were cocultured with macrophages to verify the anti-inflammatory effects of PTX3 secreted by UCB-MSCs in an inflammatory condition. The results showed that inflammatory cytokines were decreased and anti-inflammatory cytokines were increased at lower rates, with a stagnated decrease in CD11b and increase in CD163. In contrast, treatment of LPS-stimulated macrophages with recombinant PTX3 facilitated macrophage polarization, resulting in suppressed inflammation and enhanced anti-inflammation. The results indicate that PTX3 secreted by UCB-MSCs plays a pivotal role in macrophage polarization.

Next, the mechanism by which PTX3 activates macrophage polarization under inflammatory conditions was investigated. Although the cognate receptor for PTX3 is unknown, a study reported that PTX3 exerts the role of opsonin and mediates the internalization of zymosan via the Dectin-1 receptor of macrophages in inflammation [32]. Thus, based on the prediction that PTX3 interacts with Dectin-1 of macrophages, its dependency on the Dectin-1 receptor was examined. Dectin-1 expression levels were analyzed in cocultured macrophages and found to be significantly higher in the cocultured UCB-MSCs than in LPS-stimulated macrophages. Moreover, Dectin-1 expression was decreased in UCB-MSCs treated with PTX3 siRNA. This indicates an association between PTX3 and the Dectin-1 receptor of macrophages. Additionally, we examined whether Dectin-1 activated by PTX3 is involved in promoting macrophage polarization. siRNA was used to inhibit Dectin-1 expression in macrophages, followed by coculture with UCB-MSCs. The results of monitoring the level of macrophage polarization showed that polarization was decreased in macrophages when Dectin-1 was inhibited. It has been reported that Dectin-1 of macrophages reduces inflammatory cytokines while inducing anti-inflammatory cytokines via MSK1 and MSK2 phosphorylation [33]. Thus, the levels of MSK1 and MSK2 activation were measured following coculture with UCB-MSCs in an inflammatory condition. The results revealed increased levels of both proteins caused by UCB-MSCs: (1) inhibiting PTX3 secreted by UCB-MSCs led to decreased MSK1 and MSK2 expression in macrophages and (2) inhibiting Dectin-1 in macrophages led to decreased MSK1 and MSK2 expression. To further verify the association with MSK1 and MSK2, the macrophages were treated with SA747651A, an inhibitor of both MSK1 and MSK2. The results confirmed the reduction in the suppression of inflammation and enhancement of anti-inflammation in the macrophages. These results show that PTX3 secreted by USB-MSCs under an inflammatory condition activates the macrophage Dectin-1 receptor, indicating that MSK1/2 signaling positively regulates macrophage polarization. Numerous studies have reported that Dectin-1 phosphorylates the p38*α* MAPK or ERK1/2 cascade in macrophages, leading to MSK activation [33, 44–47]. MSK1/2 is also known to induce the phosphorylation of the well-known transcription factor CREB to reduce inflammatory cytokine level, while regulating the activation of anti-inflammatory cytokines [33, 46–49].

To verify the potential use of PTX3 as an efficacy marker, a well-known inflammatory disease model of BPD was used. BPD is a representative inflammatory disease [50], and preclinical studies have confirmed the therapeutic effects of injecting MSCs [28, 29, 51, 52], which is being evaluated in ongoing clinical studies in Korea and the US [53–55]. First, PTX3-silenced MSCs were injected into the disease model rats and the therapeutic effects were monitored. The survival rate of the rats was monitored until day 14 after birth, followed by analysis of MLI, M1 and M2 marker expression, Dectin-1 expression in macrophages, and residual MSCs in the lung tissue. The secretion of inflammatory and anti-inflammatory cytokines was also measured in the lung BALF. The results revealed significantly reduced therapeutic effects in PTX3-silenced UCB-MSCs, with the lowest Dectin-1 expression and residual MSCs in the lung tissue. Next, the therapeutic effects were compared by measuring the concentration of secreted PTX3. Under *in vitro* inflammatory conditions, 3 lots of UCB-MSCs (MSC5, MSC6, and MSC7) were selected based on their ability to induce PTX3 secretion and various parameters were compared between groups. MSC5 showed the lowest ability to induce PTX3 secretion and did not lead to therapeutic effects compared to the control group with respect to alveolar damage recovery, anti-inflammatory cytokine secretion, and Dectin-1 expression. In comparison, MSC6 and MSC7 induced high levels of PTX3 secretion and displayed significant therapeutic effects compared to the control group across all evaluation items. This suggests that the level of PTX3 secretion by UCB-MSCs in an inflammatory condition is an essential criterion for determining therapeutic effects.

The animal model experiment described above suggests that the PTX3 level secreted by UCB-MSCs in inflammatory conditions is a potential marker for predicting therapeutic effects. Cut-off values of PTX3 levels may also be useful in the screening of highly efficient stem cells. In agreement with this, recent studies reported that PTX3 expressed in MSCs led to therapeutic effects. In a study by Cappuzzello et al., injection of MSCs with reduced PTX3 in a skin defect model delayed wound healing compared to the control group [56]. Park et al. found that after injection of MSCs in an animal model of ischemic brain damage, tissue regeneration was promoted which decreased inflammatory reactions, with a high level of PTX3 found to be secreted by UCB-MSCs [57]. Mauri et al. injected MSCs in a mouse model of acute lung injury and observed reduced cellular fibrosis and improved lung functions, whereas the group injected with MSCs with reduced PTX3 displayed a reduced ability for lung oxygenation and the lung injury did not recover [58]. These findings suggest that PTX3 of MSCs is involved in various therapeutic effects.

## 5. Conclusion

In conclusion, UCB-MSCs under inflammatory conditions were confirmed to suppress inflammation while enhancing anti-inflammation by secreting PTX3 to activate the Dectin-1 receptor of macrophages and induce MSK1/2 signaling, thereby facilitating macrophage polarization. Furthermore, PTX3 is a potential marker for the screening of highly efficient stem cells. These findings indicate that the effects of stem cell therapy can be maximized when treating intractable inflammatory diseases.

## Data Availability

The datasets generated during the current study are available from the corresponding author on reasonable request.

## Supplementary Materials

Supplementary Materials Supplementary Figure 1: experimental scheme of MSC administration in a hyperoxic rat model. Supplementary Figure 2: silencing of PTX3 expression in UCB-MSCs. Supplementary Figure 3: macrophage polarization effect of recombinant PTX3 under LPS-induced inflammation condition. Supplementary Figure 4: effects of Dectin-1 knockdown on NR8383. Supplementary Figure 5: Dectin-1 promotes anti-inflammatory effect through MSK1/2 signaling in NR8383 under inflammation conditions. Supplementary Figure 6: silencing of PTX3 expression in UCB-MSCs. Supplementary Table 1: basic information regarding the UCB-MSCs used in this study. Supplementary Table 2: array maps of the Human Cytokine Antibody Array in Figure 2(a). Supplementary Table 3: sequences of primers used for sequencing of the indicated target genes. Supplementary Table 4: experimental design in vivo.
