# Supplementary material for: Soluble PTX3 of Human Umbilical Cord Blood-Derived Mesenchymal Stem Cells Attenuates Hyperoxic Lung Injury by Activating Macrophage Polarization in Neonatal Rat Model
Source: Stem Cells Int. 2020 Jan 23;2020:1802976. doi: 10.1155/2020/1802976 (PMC7204119; doi:10.1155/2020/1802976)
Supplement: Supplementary Materials — Supplementary Figure 1: experimental scheme of MSC administration in a hyperoxic rat model. Supplementary Figure 2: silencing of PTX3 expression in UCB-MSCs. Supplementary Figure 3: macrophage polarization effect of recombinant PTX3 under LPS-induced inflammation condition. Supplementary Figure 4: effects of Dectin-1 knockdown on NR8383. Supplementary Figure 5: Dectin-1 promotes anti-inflammatory effect through MSK1/2 signaling in NR8383 under inflammation conditions. Supplementary Figure 6: silencing of PTX3 expression in UCB-MSCs. Supplementary Table 1: basic information regarding the UCB-MSCs used in this study. Supplementary Table 2: array maps of the Human Cytokine Antibody Array in Figure 2(a). Supplementary Table 3: sequences of primers used for sequencing of the indicated target genes. Supplementary Table 4: experimental design in vivo. [file 1802976.f1.pdf]

Supplementary Materials

Soluble PTX3 of human umbilical cord blood-derived mesenchymal stem cells attenuate hyperoxic lung injury by activating macrophage polarization in neonatal rat model

Miyeon Kim, Ji Hye Kwon, Yun Kyung Bae, Soyoun Um, Gee-Hye Kim, Jueun Ha, Soo Jin Choi, Wonil Oh, Hye Jin Jin*

Biomedical Research Institute, MEDIPOST Co., Ltd., Seongnam 13494, Republic of Korea

Supplementary Figure 1. Experimental scheme of MSCs administration in a hyperoxic rat model.

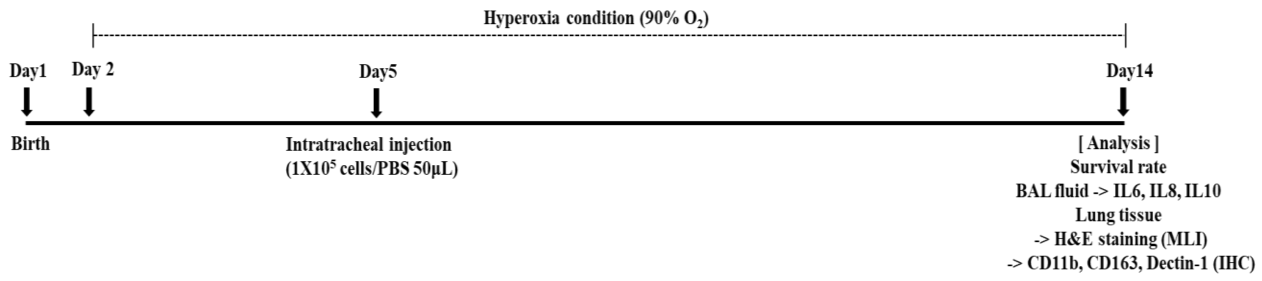

Supplementary Figure 2. Silencing of PTX3 expression in UCB-MSCs. UCB-MSCs were each transfected with scramble siRNA (Control siRNA MSC) or PTX3 siRNA (PTX3 siRNA MSC). Rat alveolar macrophages (NR8383) were stimulated with LPS and co-cultured with UCB-MSCs for 3 days. Co-culture supernatants were analyzed for PTX3 secretion of UCB-MSCs by ELISA. Expression levels were normalized to control (MΦ +L+Con siRNA MSC) with the expression levels in the control defined as 1. Error bars represent the means ± SD, n = 3 per group; ** $p < 0.01$. MΦ; macrophage, L; LPS.

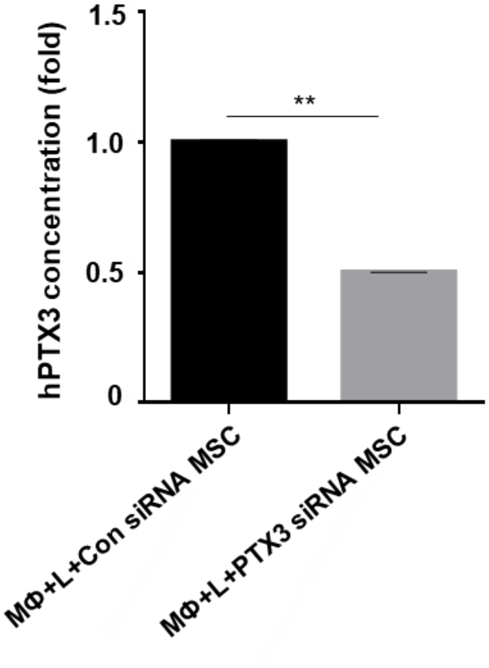

Supplementary Figure 3. Macrophage polarization effect of recombinant PTX3 under LPS-induced inflammation condition. NR8383 cells were stimulated with LPS and exposed to recombinant PTX3 for 3 days. (a) Percentage of NR8383 cells showing CD11b, CD163, and Dectin-1 expression. (b) The supernatants were analyzed for rat IL-6, IL-8, and IL-10 by ELISA. Error bars represent the means ± SD, n = 3 per group; * $p < 0.05$, ** $p < 0.01$. MΦ. $^{+}$ $p < 0.05$, $^{++}$ $p < 0.01$ vs. MΦ+L. MΦ; macrophage, L; LPS.

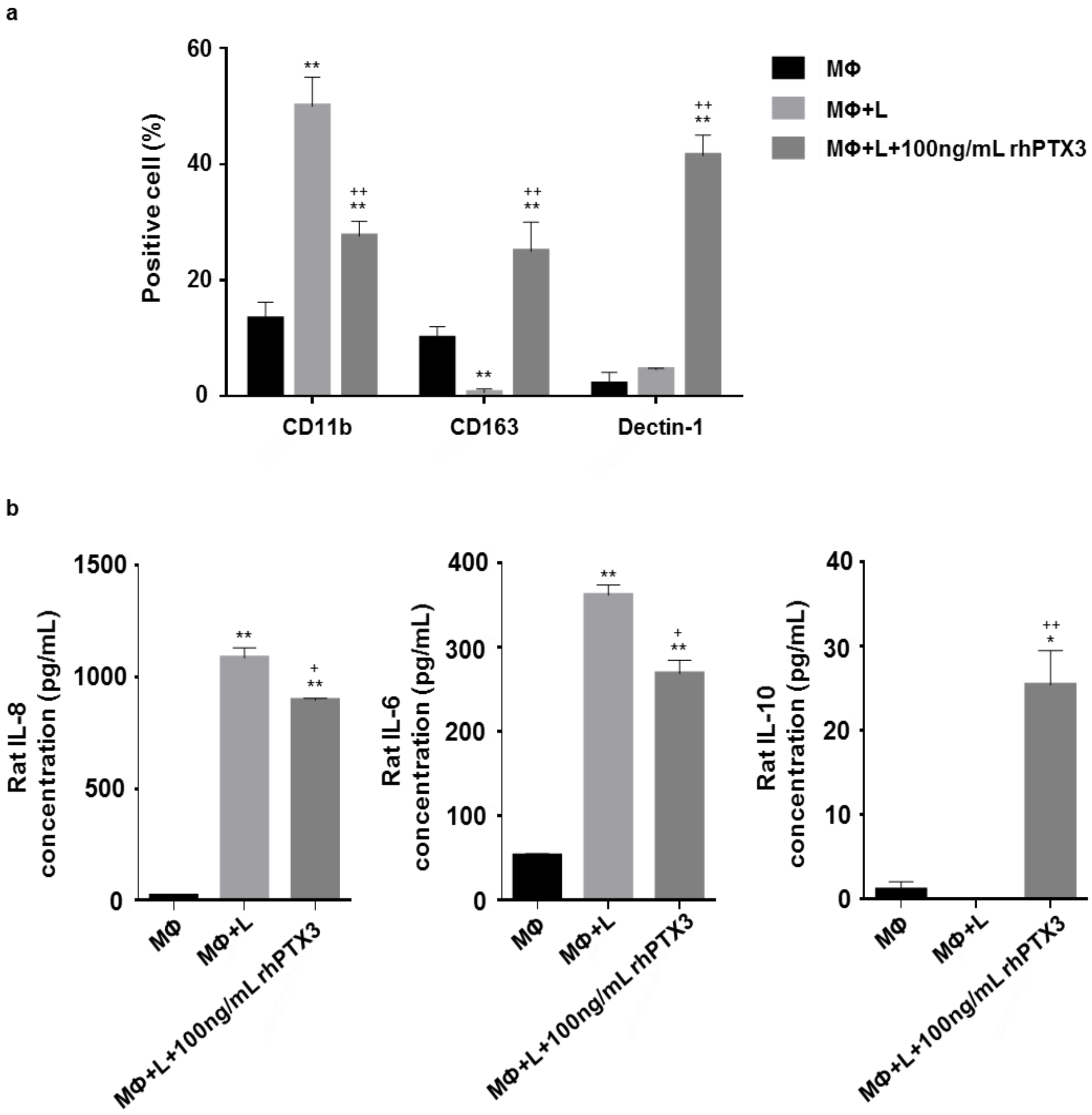

Supplementary Figure 4. Effects of Dectin-1 knockdown on NR8383 cells. NR8383 cells were transfected with scramble siRNA (Con siRNA MSC) or Dectin-1 siRNA (Dectin-1 siRNA MSC). Dectin-1 levels were measured by qPCR. Expression levels were normalized to β-actin, with the expression levels in the Con siRNA MΦ defined as 1. Error bars represent the means ± SD, n = 3 per group; ** $p < 0.01$. MΦ; macrophage.

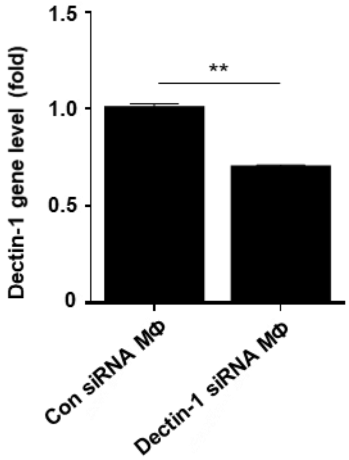

Supplementary Figure 5. Decin-1 promotes anti-inflammation effect through MSK1/2 signaling in NR8383 cells under inflammation condition. (a–b) NR8383 cells were stimulated with LPS and co-cultured with UCB-MSCs for 3 days. UCB-MSCs were pretreated with control siRNA or PTX3 siRNA. Either NR8383 was pretreated with control siRNA or Dectin-1 siRNA before co-culture. Representative immunofluorescence staining using P-MSK1 or P-MSK2 in NR8383 cells. Nuclei were stained with Hoechst 33342. Expression of P-MSK1 (a, green) and P-MSK2 (b, red) was assessed as the percentage of positively stained cells. Scale bar = 200 μm. Error bars represent the means ± SD, n = 5 per group; * $p < 0.05$, ** $p < 0.01$ vs. MΦ, ++ $p < 0.01$ vs. MΦ+L, ΔΔ $p < 0.01$ vs. MΦ+L+MSC, ◆◆ $p < 0.01$ vs. MΦ+L Con siRNA MSC, ▼▼ $p < 0.01$ vs. Con siRNA MΦ+L+MSC. (c) To test the effects of MSK1/2, we treated the NR8383 cells with SA747651A, a chemical inhibitor of MSK1/2, before co-culture. NR8383 cells were stimulated with LPS and co-cultured with UCB-MSCs for 3 days. Cell supernatants were analyzed for inflammatory cytokine (rat IL-8) or anti-inflammatory cytokine (rat IL-10) by ELISA. Error bars represent the means ± SD, n = 5 per group; ** $p < 0.01$. MΦ; macrophage, L; LPS.

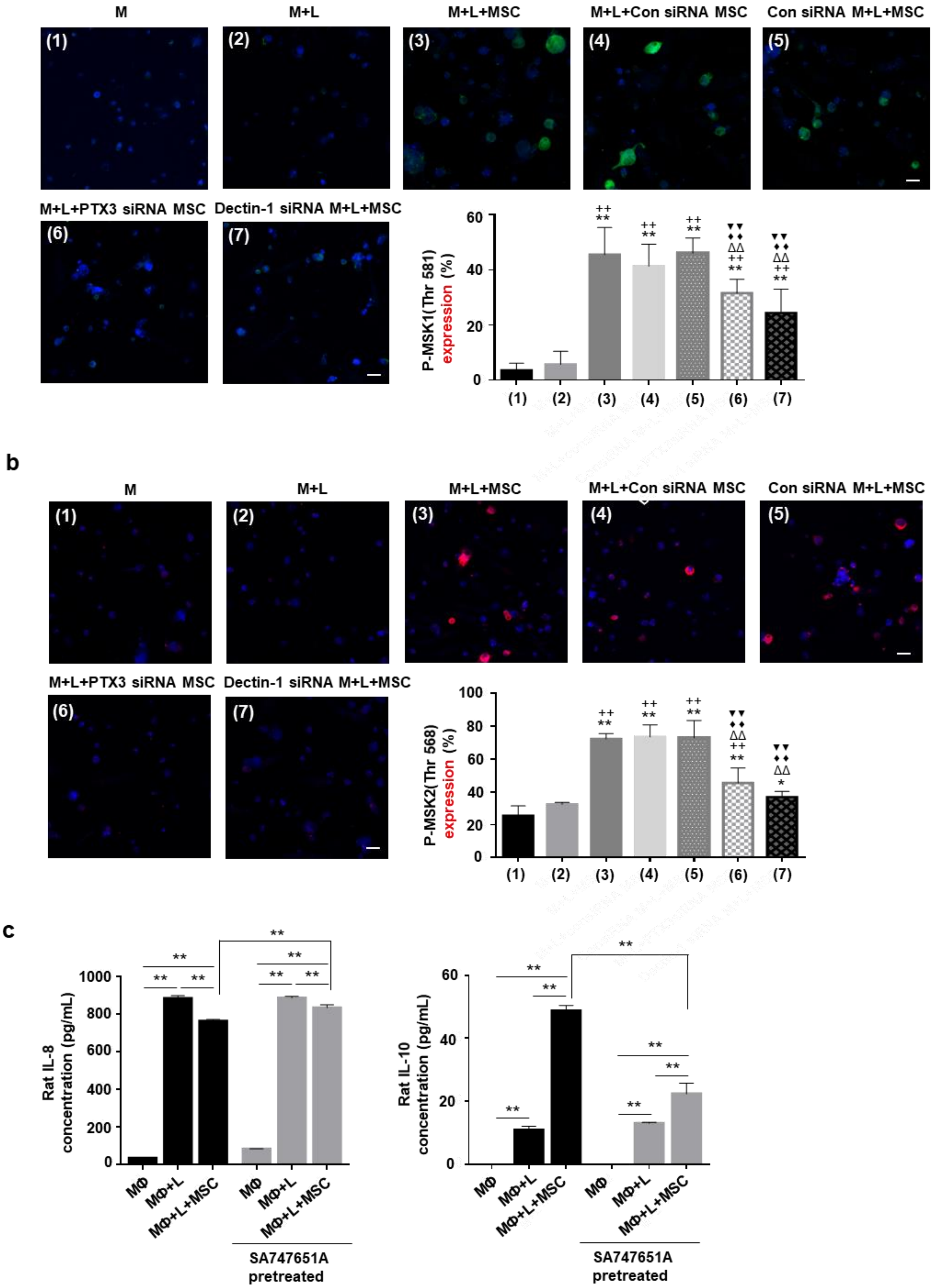

M
M+L
M+L+MSC
M+L+Con siRNA MSC
Con siRNA M+L+MSC
(1)
(2)
(3)
(4)
(5)
M+L+PTX3 siRNA MSC
Dectin-1 siRNA M+L+MSC
(6)
(7)
P-MSK1(Thr 581) expression (%)
0
20
40
60
b
M
M+L
M+L+MSC
M+L+Con siRNA MSC
Con siRNA M+L+MSC
M+L+PTX3 siRNA MSC
Dectin-1 siRNA M+L+MSC
P-MSK2(Thr 568) expression (%)
0
20
40
60
80
100
c
Rat IL-8 concentration (pg/mL)
0
200
400
600
800
1000
MΦ
MΦ+L
MΦ+L+MSC
SA747651A pretreated
Rat IL-10 concentration (pg/mL)
0
20
40
60

Supplementary Figure 6. Silencing of PTX3 expression in UCB-MSCs. UCB-MSCs were transfected with scrambled siRNA (Con siRNA) or siRNA targeting PTX3 (PTX3 siRNA). (a) siRNA-mediated suppression of human PTX3 expression was maintained for up to 13 days after transfection. The expression levels of all genes were normalized to that of β-actin in naïve cells, which was defined as 1-fold expression. Error bars represent the means ± SD, n = 3 per group; ** $p < 0.01$. (b) Concentration of human PTX3 in rat lung from transplanted UCB-MSCs at P14. Data shows human PTX3 secretion in each group by ELISA. Error bars represent the means ± SD, n = 5 per group; ** $p < 0.01$ vs. Normal, $^{++}$ $p < 0.01$ vs. BPD, $^{\Delta\Delta}$ $p < 0.01$ vs. BPD+Naïve MSC, ◆◆ $p < 0.01$ vs. BPD+Con siRNA MSC. BPD; bronchopulmonary dysplasia.

a

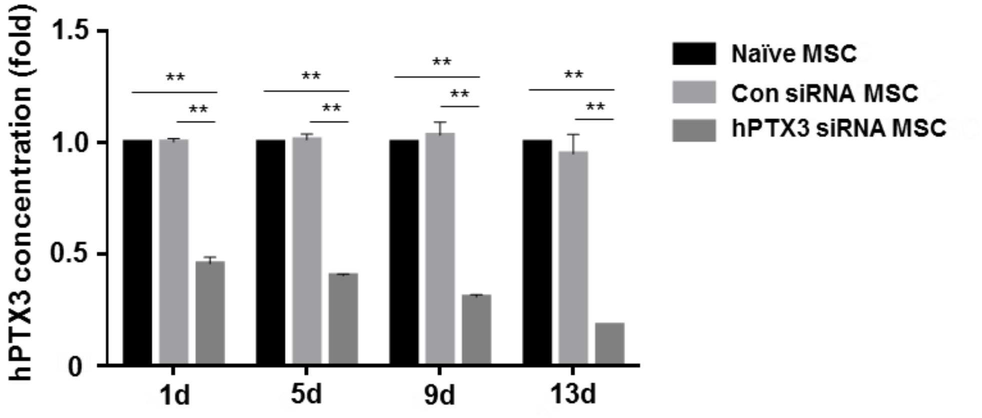

b

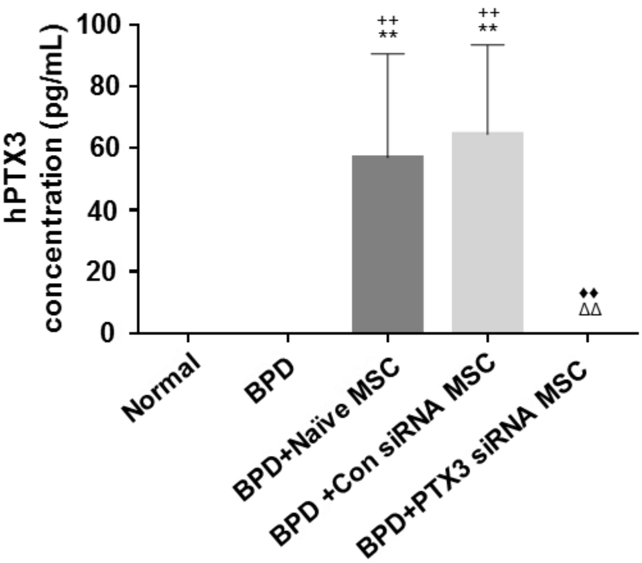

Supplementary Table 1. Basic information regarding the UCB-MSCs used in this study.

MSC	Maternal age (years)	Cell surface marker		Differentiation
		Positive	Negative	
#1	33	Pass	Pass	Pass
#2	38	Pass	Pass	Pass
#3	29	Pass	Pass	Pass
#4	33	Pass	Pass	Pass
#5	34	Pass	Pass	Pass
#6	32	Pass	Pass	Pass
#7	32	Pass	Pass	Pass

UCB-MSCs were isolated from seven independent donors (MSC #1–7). The MSC characteristics were determined by MSC marker expression or their ability to differentiate (positive: CD73, CD90, CD105, CD166 ≥85%; Negative: CD14, CD45, HLA-DR ≤1.5%; differentiation: osteogenic, chondrogenic, adipogenic).

Supplementary Table 2. Array maps of the Human Cytokine antibody Array in Figure 2a. Alternative protein names, accession numbers, and official symbols can be accessed on the Resources webpage of RayBiotech (www.raybiotech.com/I-series-507-label-based-human-array-1-glass-slide-2).

Supplementary Table 3. Sequences of primers used for sequencing of the indicated target genes

**Target gene**	**Primer sequence (5′–3′)**		**Taqman Probe #**
Human PTX3	Left	tgtatgtgaatttggacaacgaa	58
	Right	cattccgagtgctcctgac	
Rat Dectin-1	Left	cagtagtggtcgcagcagtg	82
	Right	tggttctccttatttcttgatgg	
Human β-actin	Left	ccaaccgcgagaagatga	64
	Right	ccagaggcgtacagggatag	
Rat β-actin	Left	cccgcgagtacaaccttct	17
	Right	cgtcatccatggcgaact	
Human PTX3 siRNA	i	GUGAAUUUGGACAACGAAA	
	ii	CUGCAGUGUUGGCCGAGAA	
	iii	GGUCAGGAGCACUCGGAAU	
	iv	GGAUAGUGUUCUUAGCAAU	
Rat Dectin-1 siRNA	i	GGAUAUACUCAAUUAGACU	
	ii	GAAAUAAGGAGAACCACAA	
	iii	GGUCCAUUGCGGUAGCUUU	
	iv	CCAAACUACAGGCGUCUUU	
Scramble siRNA	i	UGGUUUACAUGUCGACUAA	
	ii	UGGUUUACAUGUUGUGUGA	
	iii	UGGUUUACAUGUUUUCUGA	
	iv	UGGUUUACAUGUUUUCCUA	

Supplementary Table 4. Experimental design *in vivo*

Experimental design1			
Group	Number	MSC treat	Volume (μL)
Normal control (NC)	15	-	PBS 50
Hyperoxic lung injury (BPD)	15	-	PBS 50
BPD+naïve MSC	14	1x10^5	PBS 50
BPD+con siRNA MSC	14	1x10^5	PBS 50
BPD+PTX3 siRNA MSC	14	1x10^5	PBS 50
Experimental design 2			
Group	Number	MSC treat	Volume (μL)
Normal control (NC)	15	-	PBS 50
Hyperoxic lung injury (BPD)	15	-	PBS 50
BPD+ MSC5	15	1x10^5	PBS 50
BPD+ MSC6	15	1x10^5	PBS 50
BPD+ MSC7	15	1x10^5	PBS 50
